# Different Responsiveness of Alveolar Bone and Long Bone to Epithelial‐Mesenchymal Interaction‐Related Factor

**DOI:** 10.1002/jbm4.10382

**Published:** 2020-06-21

**Authors:** Chul Son, Moon Sil Choi, Joo‐Cheol Park

**Affiliations:** ^1^ Department of Oral Histology and Developmental Biology, School of Dentistry Seoul National University Seoul South Korea; ^2^ Department of Dental Hygiene Songwon University Gwangju South Korea

**Keywords:** epithelial‐mesenchymal interaction, neural crest, alveolar bone, long bone, bmp4

## Abstract

Alveolar bone is both morphologically and functionally different from other bones of the axial or peripheral skeleton. Because of its sensitive nature to external stimuli including mechanical stress, bone loss stimuli, and medication‐related osteonecrosis of the jaw, alveolar bone rendering is seen as an important factor in various dental surgical processes. Although multiple studies have validated the response of long bone to various factors, how alveolar bone responds to functional stimuli still needs further clarification. To examine the characteristics of bone in vitro, we isolated cells from alveolar, femur, and tibia bone tissue. Although primary cultured mouse alveolar bone‐derived cells (mABDCs) and mouse long bone‐derived cells (mLBDCs) exhibited similar osteoblastic characteristics, morphology, and proliferation rates, both showed distinct expression of neural crest (NC) and epithelial–mesenchymal interaction (EMI)‐related genes. Furthermore, they showed significantly different mineralization rates. RNA sequencing data demonstrated distinct transcriptome profiles of alveolar bone and long bone. Osteogenic, NC‐, and EMI‐related genes showed distinct expression between mABDCs and mLBDCs. When the gene expression patterns during osteogenic differentiation were analyzed, excluding several osteogenic genes, NC‐ and EMI‐related genes showed different expression patterns. Among EMI‐related proteins, BMP4 elevated the expression levels of osteogenic genes, *Msx2*, *Dlx5*, and *Bmp2* the most, more noticeably in mABDCs than in mLBDCs during osteogenic differentiation. In in vivo models, the BMP4‐treated mABDC group showed massive bone formation and maturation as opposed to its counterpart. Bone sialoprotein expression was also validated in calcified tissues. Overall, our data suggest that alveolar bone and long bone have different responsiveness to EMI by distinct gene regulation. In particular, BMP4 has critical bone formation effects on alveolar bone, but not on long bone. © 2020 The Authors. *JBMR Plus* published by Wiley Periodicals, Inc. on behalf of American Society for Bone and Mineral Research.

## Introduction

Teeth have many mechanical stressors from a variety of external forces, including mastication, tooth brushing, and injury.^(^ ^1^ ^)^ Against these stressors, periodontium—tissue covering the tooth root—supports the tooth.^(^ ^2^ ^)^ Among the tissues that makeup periodontium, alveolar bone has interesting conformation changes sensitive to external forces. At times, such bone sensitivity allows rearrangement of the tooth to its proper site.^(^ ^3^ ^)^ The condition of alveolar bone is clinically significant in many cases, including periodontitis and dental implant surgery.^(^ ^4^, ^5^ ^)^ However, the development and underlying genetic characteristics of alveolar bone still require further investigation.

To understand the genetic and molecular makeup of alveolar bone, we selected long bone for our comparison because of its pre‐existing extensive research. When compared, alveolar and long bone showed different responsiveness. Generally, bone is a dynamic tissue that responds to environmental stimuli; for example, reduction of mechanical loading on bone results in bone loss.^(^ ^6^ ^)^ Although bone does generally respond to environmental stimuli, alveolar bone is especially sensitive to mechanical forces.^(^ ^3^ ^)^ Such sensitivity has been taken advantage of in orthodontic procedures. Another difference in responsiveness can be seen in osteoporosis treatment. When treated with osteoporosis drugs, long bone has shown positive results. In contrast, alveolar bone has shown detrimental effects from the same drugs.^(^ ^7^, ^8^ ^)^ These different phenomena infer that fundamental differences exist between alveolar bone and long bones.

Previous studies have suggested that the developmental process of alveolar and long bone is different. Epithelial–mesenchymal interaction (EMI), especially, is known to play an essential role in organ development, including bone.^(^ ^9^ ^)^ Early development of both alveolar bone and long bone is closely related to EMI. For long bone, the formation of a limb bud emerges from the interaction between apical ectodermal ridge, a specialized epithelium, and mesenchyme, which is derived from lateral plate mesoderm.^(^ ^10^ ^)^ For alveolar bone, the development of periodontium results from the interaction between dental epithelium and ectomesenchyme—a form of mesenchyme that migrates from the neural crest (NC).^(^ ^11^ ^)^ Moreover, recent studies have shown the linkage between alveolar bone remodeling and epithelial cell rests of Malassez in adult periodontium.^(^ ^12^ ^)^ Using these differences in EMI interaction, we attempt to understand the uniqueness of alveolar bone by using developmental differences.

We found distinct genetic profiles of EMI‐related genes, NC‐related genes, and osteogenic genes by investing transcriptome data in mouse primary cultured alveolar bone‐derived cells (mABDCs) and long bone‐derived cells (mLBDCs). The EMI‐related factors produced by dental epithelium showed different effects in mABDCs and mLBDCs. Finally, our results provided evidence that BMP4 regulates bone formation differently in alveolar bone and long bone.

## Materials and Methods

### Tissue preparation and histology

All animal experiments followed protocols approved by the Institutional Animal Care and Use Committee of Seoul National University (SNU‐160509‐6). The head and long bone dissected from C57BL/6 male mice were fixed in 4% paraformaldehyde (PFA; Sigma‐Aldrich, St. Louis, MO, USA) at 4°C overnight and decalcified in a 10% EDTA (Georgiachem, Suwanee, GA, USA) for 2 weeks at room temperature. Embedded tissues were sectioned at a thickness of 5 μm. To evaluate histologic findings, sections were stained with H&E (Vector Labs, Burlingame, CA, USA). For histomorphometric analysis, an optical microscope (BX50; Olympus Co., Tokyo, Japan) connected to a computer and charge‐coupled device (CCD) camera (DP71; Olympus Co.) and an adaptor (U‐TV0.63XC; Olympus Co.) was used to take images of the samples. Image analysis of new bone and marrow formation was performed with analySIS LS Starter (Olympus Soft Imaging Solutions GmbH, Muenster, Germany).

### Primary cell culture

Alveolar and long bones were collected from 7‐day‐old C57BL/6 mice. After euthanasia, the alveolar, tibia, and femur bones were removed and cells were released from the matrix using 1‐mL digestion medium (0.1% collagenase type I [GIBCO, Waltham, MA, USA], 0.2% Dispase [GIBCO] diluted in α‐MEM [GIBCO]) at 37°C with shaking. After 5‐min digestion, the first fraction was collected and discarded, and four subsequent fractions were collected every 10 min for 40 min and pooled. Cells were plated in α‐MEM containing 10% FBS (GIBCO) and antibiotic–antimycotic reagents (GIBCO) in 10‐cm dishes. Cells were allowed to proliferate until 80% to 90% confluency, seeded into 6‐cm dishes, and cultured until confluency (day 0 time point). Osteogenic differentiation was induced with 5mM sodium beta‐glycerophosphate (Sigma‐Aldrich) and 50 μg/μL ascorbic acid (Amresco, Solon, OH, USA) in proliferation medium. Differentiation medium was changed every 2 to 3 days. Samples were collected at 0, 4, 7, 10, and 14 days by thoroughly rinsing the wells with PBS, and stored in −80°C.

### Real‐time PCR analysis

Total RNA was extracted from cells with Tri reagent according to the manufacturer's instructions (MRC, Cincinnati, OH, USA). Total RNA (3 μg) was reverse transcribed using Superscript IV Reverse Transcriptase (Invitrogen, Carlsbad, CA, USA) and oligo (dT) primers (Invitrogen). One μL of the RT product was PCR‐amplified using the primer pairs. For real‐time PCR, the specific primers for *Bsp, Osteocalcin* (Oc), *Alp, Runx2, Osx, Bmp2, Bmp4, Nfic, Cpne7, Msx1, Msx2, Dlx5*, and *Dmp1* were synthesized as listed in Supplementary Table S1. Real‐time PCR was performed on a Step One Plus sequence detection system (Applied Biosystems, Foster City, CA, USA) using iTaq Universal SYBR Green Supermix (Bio‐Rad Laboratories, Hercules, CA, USA) according to the manufacturer's instructions. PCR conditions were 40 cycles at 95°C for 15 s and 60°C for 1 min. All reactions were performed in triplicate, and PCR product levels were normalized to that of the housekeeping gene, glyceraldehyde 3‐phosphate dehydrogenase (Gapdh). Relative changes in gene expression were calculated using the comparative threshold cycle (C_T_) method.

### Alizarin Red staining for mineralized matrix

Cells were seeded into 6‐cm culture dishes at a density of 0.8 × 10^5^ cells per dish. Osteogenic differentiation was induced after each dish reached confluency. At a certain time point, cells were fixed with 4% PFA overnight at 4°C, and stained with 40mM Alizarin Red S (Sigma‐Aldrich), pH 4.2 for 30 min at room temperature. For the quantification of mineralized matrix in culture, Alizarin Red stain was eluted using 0.5 mL of 5% sodium dodecyl sulfate (Amresco) in 0.5 N HCl solution, with shaking for 30 min; the absorbance of the eluted dye was measured at 405 nm.

### Cell proliferation assay

Cells were seeded in a 96‐well plate at 5000 per well in a CO_2_ incubator at 37°C in triplicates; the samples were processed for MTT [3‐(4,5‐dimethyl‐2‐thiazolyl)‐2,5‐diphenyl‐tetrazolium bromide] assay at day 0, 1, 2, and 3. Then 150 μL of 5‐g/L MTT solution were added to each well for 2 hours at 37°C. The cells were then lysed in DMSO (Duksan Chemical Co., Ltd., Yongin, Gyeonggi, Republic of Korea), and absorbance at 570 nm was determined with a microplate reader.

### mRNA‐seq data

To construct cDNA libraries with the TruSeq RNA library kit, 1 μg of total RNA was used. The protocol consisted of polyA‐selected RNA extraction, RNA fragmentation, random hexamer primed reverse transcription, and 100 nt paired‐end sequencing by Illumina HiSeq2500 (Illumina, San Diego, CA, USA). The libraries were quantified using qPCR according to the qPCR Quantification Protocol Guide and qualified using an Agilent Technologies 2100 Bioanalyzer (Agilent Technologies, Santa Clara, CA, USA).

We processed reads from the sequencer and aligned them to the *Mus musculus (mm10)* using Tophat v2.0.13.^(^ ^13^ ^)^ Tophat incorporates the Bowtie v2.2.3^(^ ^14^ ^)^ algorithm to perform the alignment and mapping. We used Cufflinks for transcript assembly and abundance estimation.^(^ ^15^ ^)^ After aligning reads to the genome, Cufflinks v2.2.1 was used to assemble aligned reads into transcripts and to estimate their abundance. The transcript counts in isoform level were calculated, and the relative transcript abundances were measured in FPKM (fragments per kilobase of exon per million fragments mapped) from Cufflinks. Gene‐level expression values were also calculated from the transcript counts. We excluded genes with zeroed FPKM values >1 for total samples. We added 1 with the FPKM value of the filtered gene to facilitate log2 transformation. Filtered data were transformed by logarithm and normalized by the quantile normalization method.

We used a multidimensional scaling method to visualize the similarities among samples. Hierarchical clustering analysis also was performed, using complete linkage and Euclidean distance as a measure of similarity to display the expression patterns of differentially expressed genes (DEG) that are satisfied with |fold change| ≥ 2. Biologically gene functional annotation analysis for the DEG list was performed using the DAVID tool (http://david.abcc.ncifcrf.gov/) to understand biological meanings behind large list of genes.^(^ ^16^ ^)^

All data analyses and visualizations of differentially expressed genes were conducted using R (R Foundation for Statistical Computing, Vienna, Austria; https://www.r-project.org/).

### Western blot analysis

The whole‐cell lysates of cells were harvested using a lysis buffer consisting of 50mM Tris–HCl, pH 7.4, 150mM NaCl, 1% Nonidet P‐40, 1mM EDTA, and 1mM phenylmethylsulfonylfluoride supplemented with protease inhibitors (Roche Molecular Biochemicals, Mannheim, Germany). Following centrifugation at 13,000*g* for 30 min, the supernatant was collected for analysis. Protein concentrations were determined using the DC protein assay system (Bio‐Rad Laboratories, Hercules, CA, USA). Proteins (20 μg) were resolved using 10% polyacrylamide gel electrophoresis and transferred to a polyvinylidene fluoride (PVDF) membrane (Merck Milipore, Billerica, MA, USA). The PVDF membrane was blocked with Tris‐buffered saline Tween‐20 (TBST; 20mM Tris‐buffered saline, pH 7.4; Tech&Innovation, Gangwon, Korea), and 0.1% Tween‐20; Amresco) buffer containing 5% nonfat dry milk (Becton Dickinson, BD; Franklin Lakes, NJ, USA) for 1 hour at room temperature. The blots were then washed and incubated with the indicated antibodies for overnight at 4°C with gentle shaking. Affinity‐purified rabbit polyclonal anti‐CPNE7, anti‐NFIC, and anti‐BSP (bone sialoprotein) antibodies were produced as described previously.^(^ ^17^, ^18^ ^)^ The anti‐ALP (sc‐30203), anti‐RUNX2 (sc‐10758), and anti‐GAPDH (sc‐25778) antibodies were purchased from Santa Cruz Biotechnology (Santa Cruz, CA, USA). The anti‐OC (ab223692), anti‐BMP2 (ab14933), anti‐BMP4 (ab39973), anti‐MSX1 (ab174207), anti‐MSX2 (ab223692), and anti‐DLX5 (ab64827) antibodies were purchased from Abcam (Cambridge, MA, USA). The anti‐OSX (PA5‐40509) was purchased from Invitrogen. Blots were washed three times for 10 min each in TBST, followed by incubation with anti‐rabbit or anti‐mouse immunoglobulin G conjugated to horseradish peroxidase (Santa Cruz Biotechnology) in TBST for 1 hour at room temperature. After washing three times in TBST, the blots were analyzed using an enhanced chemiluminescence reagent (Dogen, Cambridge, MA, USA) according to the manufacturer's guidelines. Protein loading was assessed by the expression of GAPDH.

### Ectopic transplantation in vivo and histological analysis

The primary cultured mouse bone‐derived cells (1 × 10^6^) were mixed with 100‐mg hydroxyapatite/tricalcium phosphate (HA/TCP) ceramic powder (Zimmer, Warsaw, IN, USA) alone or with BMP4 (5 μg; Peprotch, Rocky Hill, NJ, USA) in a 0.5% fibrin gel, and then transplanted s.c. into immunocompromised mice (NIH‐bg‐ nu‐xid; Harlan Laboratories, Indianapolis, IN, USA) for 6 and 12 weeks.

For histomorphometric analysis of newly formed mineralized tissue, samples were harvested and fixed in 4% PFA, decalcified in 10% EDTA (pH 7.4), embedded in paraffin, and stained with H&E, Masson's trichrome (Polysciences Inc., Warrington, PA, USA), or processed for immunohistochemistry. For immunohistochemistry, proteins were detected with anti‐BSP^(^ ^17^ ^)^ at a dilution of 1:100 as the primary antibody and a biotin‐labeled goat anti‐rabbit IgG (Vector Labs) as the secondary antibody. Tartrate‐resistant acid phosphatase staining was performed. The total mineralized area among the regenerated bone‐ and marrow‐like tissue was analyzed using the LS starter program (Olympus Soft Imaging Solutions).

### Statistical analysis

All values are expressed as mean ± SD of at least three independent experiments. The Student's *t* test was used for comparison between two groups. The two‐way ANOVA was used for comparison of more than three groups. Differences were considered statistically significant at *p < 0.05 and **p < 0.005.

## Results

### Comparing the development of alveolar bone and long bone

Prior to comparing long and alveolar bone, alveolar bone development stages were explored throughout the tooth development stages of the molar. At the bud stage, embryonic day 12 (E12), multiple mesenchymal stem cells beneath the tooth bud were condensed to form basal bone (Supplementary Fig. S1 *A*). At the bud‐to‐cap transition, E14, mineralized bone was discovered around the site where basal bone usually forms. When enamel organ begins to form cervical loops, dental follicle near the cervical loop started to form alveolar bone (Supplementary Fig. S1 *B*). At the cap‐to‐bell stage, E16 and E19, alveolar bone grew more actively near the developing tooth. Incisors began to be seen beneath the bone that covers the molar root (Supplementary Fig. S1 *C*,*D*,*E*). At the hard tissue formation, postnatal day 7 (PN7), molars and incisors were almost fully covered by alveolar bone. Specifically, when the distance between outer and inner dental epithelium became closer, the molar‐region alveolar bone almost fully covered the crown region (Supplementary Fig. S1 *F*). At the root formation stage, PN21, the majority of the bones were well‐matured, while small amounts of dental epithelial‐derived tissues remained in the periodontal ligaments (Supplementary Fig. S1 *G*).

Long bone was analyzed with the femur, specifically the diaphysis region. At E12 and E14, most of the hind limbs were formed with cartilage (Supplementary Fig. S1 *H*,*I*). At E16 and E19, diaphysis parts transformed to bone tissue (Supplementary Fig. S1 *J*, *K*). At PN7, bone and marrow cavity became distinguishable (Supplementary Fig. S1 *L*). At PN21, cortical bone and marrow cavity were well‐matured (Supplementary Fig. S1 *M*). These data elucidate that alveolar bone development begins at the bud‐to‐cap stage and is most actively formed at the hard tissue formation. Simultaneously, long bone was discovered to actively form at the same stage. During the development, epithelial tissue was located more closely and for a longer duration to alveolar bone than to long bone.

### Distinct characteristics of primary mouse alveolar bone‐ and long bone‐derived cells

To analyze the characteristics of each bone cell in vitro, we harvested bone cells from each tissue. Alveolar bone‐ and long bone‐derived cells were isolated at the hard tissue formation stage, when both bone formations were most active (Supplementary Fig. S1 *F*,*L*). mABDCs and mLBDCs were explanted from each of the bone pieces; their morphology looked similar (Supplementary Fig. S2 *A*,*B*,*C*,*D*). Proliferative activity was similar in both bone‐derived cells (Supplementary Fig. S2 *E*). To confirm their osteoblastic characteristic, we compared both bone‐derived cells with nonosteogenic cells, specifically mouse primary dermal fibroblasts (mDFs) from the ventral skin. *Bsp* was highly expressed in both bone‐derived cells when compared with mDFs. Both bone‐derived cells manifested mineralized nodule formation after osteogenic differentiation, but not in mDFs (Supplementary Fig. S2 *F*,*G*). These results indicate that mABDCs and mLBDCs have osteoblastic characteristics and similar proliferative activity and morphology.

**Figure 1:**
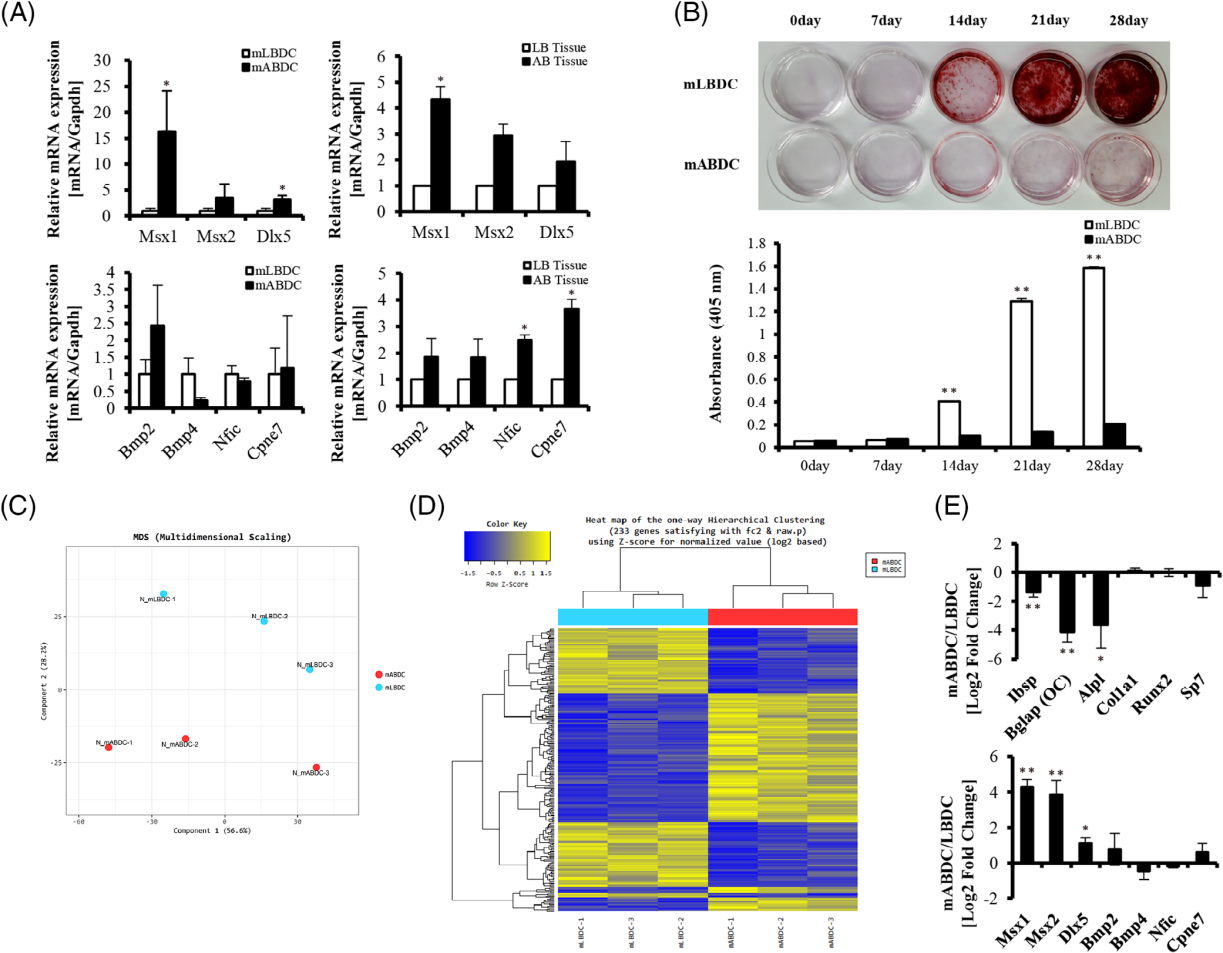
Characteristics of alveolar bone and long bone. (*A*) NC‐ and EMI‐related gene expression of confluent, primary cultured mouse bone‐derived cells, and bone tissues analyzed by qPCR. All values are normalized to the *Gapdh*. (*B*) ARS in both bone‐derived cells during osteogenic differentiation for 28 days. (*C–E*) Comparing expression profiles between mABDCs and mLBDCs using transcriptome sequencing data. (*C*) Multidimensional scaling plot. (*D*) Heat map for hierarchical clustering. (*E*) Relative gene expressions from set of osteogenic, NC‐, and EMI‐related genes. Sequencing data analyzed from triplicated RNA sequencing data of mABDC and mLBDC samples. All statistical analysis performed by Student's *t* test, *n* = 3, *p < 0.05, **p < 0.005. AB tissue = alveolar bone tissue; Alpl = alkaline phosphatase; ARS = Alizarin Red S staining; Bglap = osteocalcin; Dlx = distal‐less homeobox; Bmp = bone morphogenetic protein; Cpne7 = copine‐7; EMI = epithelial–mesenchymal interaction; Ibsp = bone sialoprotein; LB tissue = long bone tissue; mABDCs = mouse alveolar bone‐derived cells; mLBDCs= mouse long bone‐derived cells; Msx = msh homeobox; NC = neural crest; Nfic = nuclear factor I‐C; Runx2 = runt‐related transcription factor 2; Sp7 = osterix; Col1a1 = collagen type I alpha 1 chain.

To investigate whether the NC‐ and EMI‐related gene were differently regulated between two tissues, we measured the mRNA expression level of the Msx and Bmp family, *Dlx5, Nfic*, and *Cpne7* genes in cells and tissues. The expression levels of *Msx* and *Dlx* genes were higher in cell and tissue of alveolar bone than in long bone. Interestingly, the expression levels of *Bmp*, *Nfic*, and *Cpne7* genes were not consistent between alveolar bone and long bone. Contrary to *Nfic* and *Cpne7, Bmp2* and *Bmp4* mRNA expressions were distinct in mABDCs and mLBDCs. However, the expression level of all EMI‐related genes was higher in alveolar bone than in long bone tissue (Fig. 1*A*). The mineralization capacities were also different in mABDCs and mLBDCs. Mineralized nodule formation was more elevated in mLBDCs than in mABDCs (Fig. 1*B*). Therefore, we focused on gene expression at the genome level to find more evidence of different characteristics. The expression profiles between alveolar bone and long bone were compared using transcriptome sequencing data. The multidimensional scaling plot and heat map for hierarchical clustering showed the distinct transcriptome profile between mABDCs and mLBDCs (Fig. 1*C*,*D*). Moreover, gene ontology about biological process, molecular function, and cellular component; and KEGG pathway analysis for functional studies also showed different characteristics (Supplementary Fig. S3). To analyze the precise gene expression change, we focused on the classes of genes related to osteogenic differentiation, NC and EMI. Most of the osteogenic genes were more highly expressed in mLBDCs than in mABDCs. NC‐ and EMI‐ related genes showed a consistent pattern between the two types of bone cells (Fig. 1*E*). To verify the origin of bone, cranial NC cells and derivative marker expression were also analyzed (Supplementary Fig. S4). Our results indicate that gene expression profiles of alveolar bone and long bone are different. Alveolar bone expresses higher levels of NC‐related genes than long bone. Meanwhile, EMI‐related gene expression profiles were different between the two different bones, both at the cellular and tissue levels.

**Figure 2:**
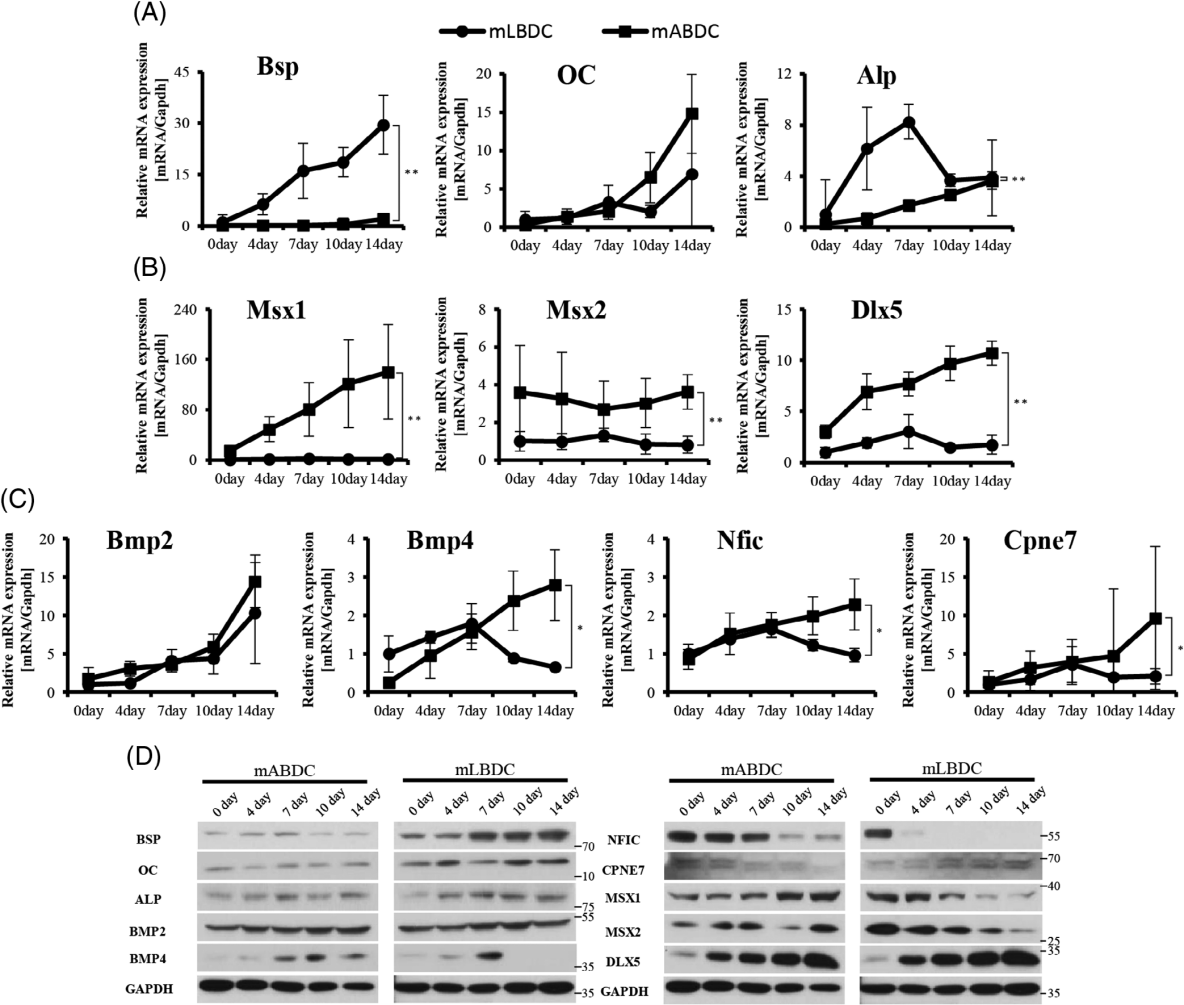
Expression levels of osteogenic, NC‐, and EMI‐related genes during osteogenic differentiation in mLBDCs and mABDCs. (*A*) Osteogenic genes, *Bsp, OC*, and *Alp* expression levels observed during differentiation. (*B*) NC‐related genes, *Msx1, Msx2*, and *Dlx5* expression levels observed during differentiation. (*C*) EMI‐related genes, Bmp family, *Nfic*, and *Cpne7* expression levels observed during differentiation. Real‐time PCR values are normalized to the *Gapdh*. (*D*) All values were also evaluated by protein expression level. GAPDH was used as internal control. Molecular weight markers were mentioned beside. Differentiation of mLBDCs and mABDCs for 14 days and analyzed by qPCR and Western blot. All statistical analysis performed by two‐way ANOVA, *n* = 3, *p < 0.05, **p < 0.005. Bsp = bone sialoprotein; Alp = alkaline phosphatase; Dlx = distal‐less homeobox; Bmp = bone morphogenetic protein; Cpne7 = copine‐7; EMI = epithelial–mesenchymal interaction; mABDCs = mouse alveolar bone‐derived cells; mLBDCs= mouse long bone‐derived cells; Msx = msh homeobox; Nfic = nuclear factor I‐C; OC = osteocalcin.

To compare the gene regulation in mABDCs and mLBDCs during osteogenic differentiation, we analyzed mRNA expression patterns of osteogenic, NC‐, and EMI‐related genes during osteogenic differentiation. *Bsp, OC*, and *Bmp2* showed continuously increased expression patterns in both. Meanwhile, NC‐related genes, EMI‐related genes, and *Alp* showed different expression patterns between mABDCs and mLBDCs. In mLBDCs, the expression pattern of the genes gradually increased during early differentiation and decreased during late differentiation. In mABDCs, however, the expression progressively increased during differentiation. Only *Msx2* expression gradually decreased during early differentiation and increased during late differentiation (Fig. 2*A*–*C*). Protein expression levels showed consistently different patterns, excluding DLX5 (Fig. 2*D*). These data could suggest that gene expression in mABDCs and mLBDCs were different during osteogenic differentiation.

### Different effects of dental epithelial secreted proteins in mABDCs and mLBDCs

**Figure 3:**
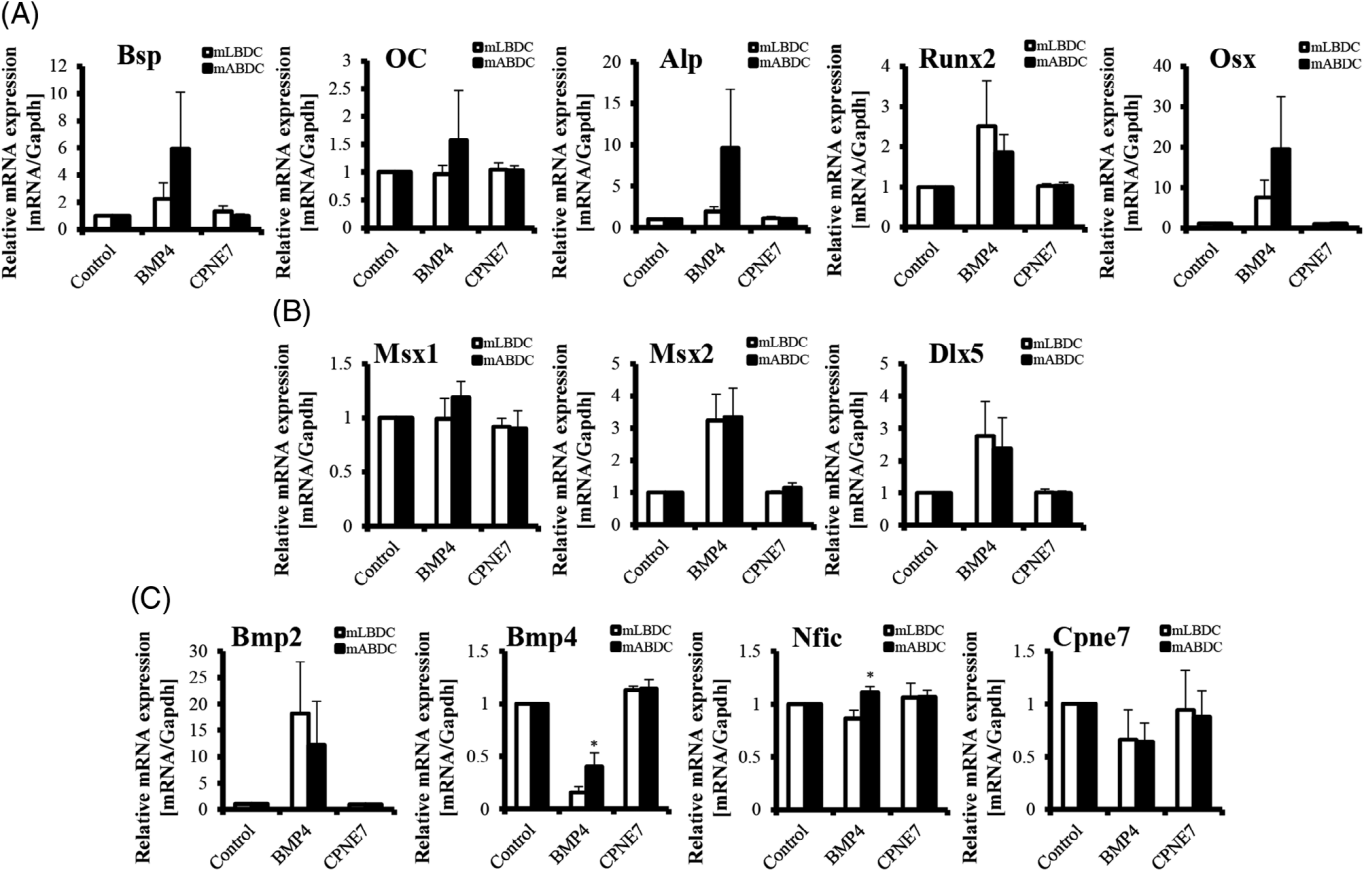
Expression levels of osteogenic, NC‐, and EMI‐related genes in mLBDCs and mABDCs after EMI‐related proteins treatment. (*A*) Expression levels of osteogenic genes. (*B*) Expression levels of NC‐related genes. (*C*) Expression levels of EMI‐related genes. Real‐time PCR values are normalized to the *Gapdh*. Cells were treated with 100 ng/mL proteins for 48 hours and analyzed by qPCR. All statistical analysis performed by Student's *t* test, *n* = 3, *p < 0.05. Bsp = bone sialoprotein; Alp = alkaline phosphatase; Dlx = distal‐less homeobox; Bmp = bone morphogenetic protein; Cpne7 = copine‐7; EMI = epithelial–mesenchymal interaction; mABDCs = mouse alveolar bone‐derived cells; mLBDCs= mouse long bone‐derived cells; Msx = msh homeobox; Nfic = nuclear factor I‐C; OC = osteocalcin; Osx = osterix; Runx2 = runt‐related transcription factor 2.

We found different expressions of osteogenic and EMI‐related genes in mABDCs and mLBDCs. To find the effect of EMI on other genes—especially in alveolar bone—we treated EMI‐related protein, which is known to be secreted from dental epithelium, to mABDCs and mLBDCs. We analyzed mRNA expression levels of the osteogenic, NC‐, and EMI‐related genes after protein treatment. Expression of most osteogenic genes was elevated in both bone‐derived cells in the BMP4‐treatment group, except *OC*, which was only elevated in mABDCs. In BMP4‐treated groups, the rate of increase in most osteogenic gene expressions was higher in mABDCs than in mLBDCs. Meanwhile, *Runx2* was more elevated in mLBDCs (Fig. 3*A*). NC‐related genes, *Msx2* and *Dlx5*, were upregulated in the BMP4‐treatment group (Fig. 3*B*). EMI‐related genes showed diverse results. *Bmp2* was highly elevated in the BMP4‐treated group. But Bmp4 was downregulated in BMP4‐treated groups. *Nfic* expression showed the opposite effects of BMP4 in each cell. *Cpne7* expression was slightly repressed in all protein‐treated groups (Fig. 3*C*). The data indicate that BMP4 regulates most osteogenic and NC‐related genes more than CPNE7 in both bone‐derived cells. The effects seemed more efficient in mABDCs than in mLBDCs.

**Figure 4:**
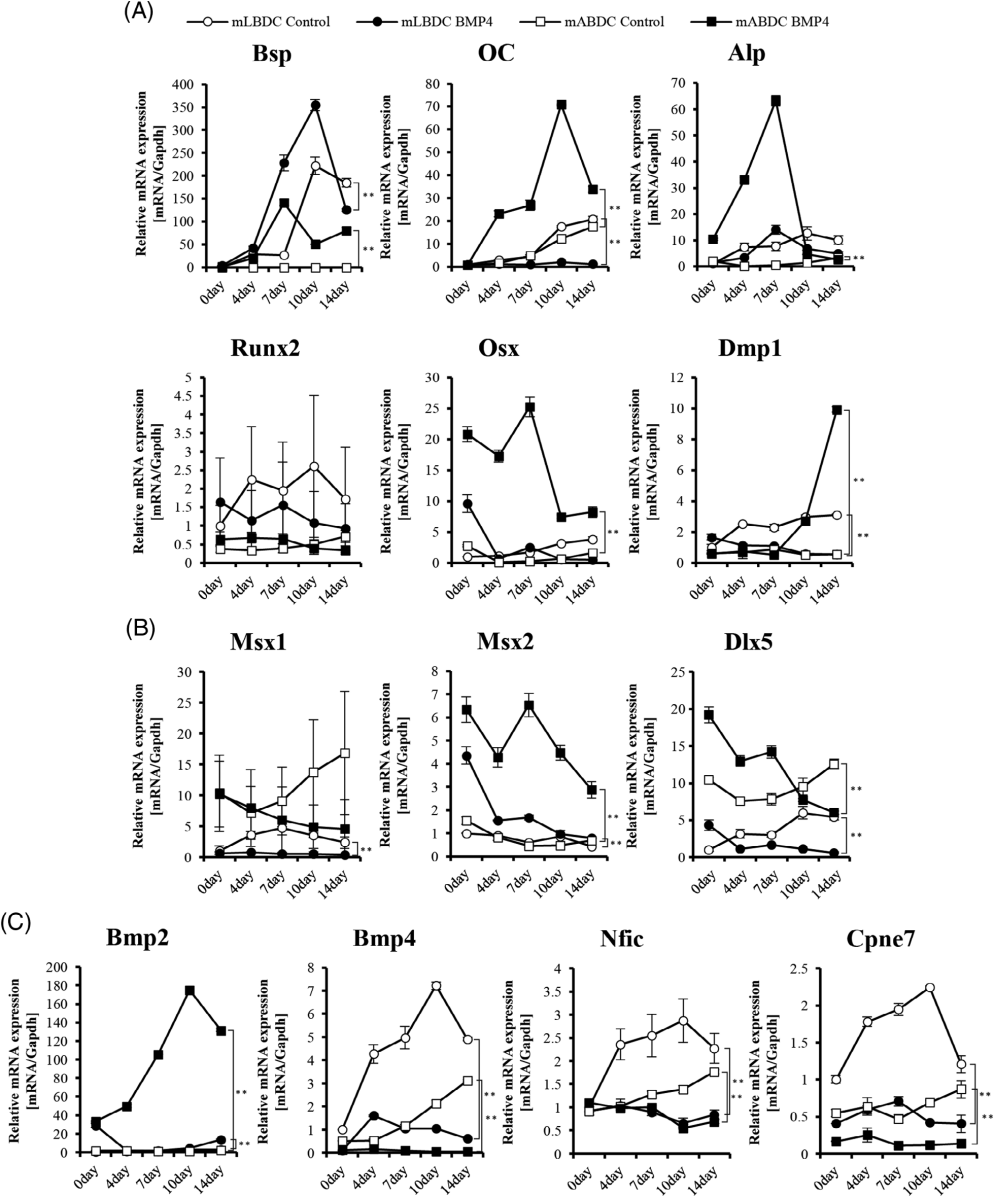
Expression levels of osteogenic, NC‐, and EMI‐related genes in BMP4‐treated mLBDCs and mABDCs during osteogenic differentiation. (*A*) Expression levels of osteogenic genes. (*B*) Expression levels of NC‐related genes. (*C*) Expression levels of EMI‐related genes. Real‐time PCR values are normalized to the *Gapdh*. Cells were treated with 100 ng/mL BMP4 proteins during differentiation and analyzed by qPCR. All statistical analysis performed by two‐way ANOVA, *n* = 3, *p < 0.05, **p < 0.005. Bsp = bone sialoprotein; Alp = alkaline phosphatase; Dlx = distal‐less homeobox; Bmp = bone morphogenetic protein; Cpne7 = copine‐7; EMI = epithelial–mesenchymal interaction; mABDCs = mouse alveolar bone‐derived cells; mLBDCs= mouse long bone‐derived cells; Msx = msh homeobox; Nfic = nuclear factor I‐C; OC = osteocalcin; Osx = osterix; Runx2 = runt‐related transcription factor 2; Dmp1 = dentin matrix protein 1.

BMP4 showed a more dynamic effect in bone‐derived cells from the different EMI‐related secretion proteins from epithelial tissue. Specially, *Msx2* and *Dlx5* were also affected by that protein. To investigate whether BMP4 regulates genes during osteogenic differentiation, respectively, in mABDCs and mLBDCs, we analyzed mRNA expression in cells. In mABDCs, osteogenic genes were elevated in BMP4‐treated groups during early differentiation stages. Late osteogenic markers, *OC, Osx*, and *Dmp1*, also showed higher expression in the BMP4‐treated group than in the control group. In BMP4‐treated mLBDCs, all of the osteogenic genes were downregulated, but *Bsp* was upregulated during differentiation. Even the rate of increase in *Bsp* expressions was higher in BMP4‐treated mABDCs than in mLBDCs (Fig. 4*A*). Some NC‐ and EMI‐related genes, *Msx2* and *Dlx5*, were also highly expressed in both BMP4‐treated mABDCs. The mRNA expression pattern of *Dlx5* was downregulated during late osteogenic differentiation in BMP4‐treated groups. Similar downregulated patterns were found in other osteogenic genes in mABDCs (Fig. 4*B*). In EMI‐related genes, *Bmp2* was extremely elevated; however, *Bmp4* was downregulated in both BMP4‐treated cells similar to the nondifferentiated status results. Both *Nfic* and *Cpne7* were suppressed during osteogenic differentiation in BMP4‐treated groups (Fig. 4*C*). These results show that BMP4 significantly induces osteogenic and NC‐related genes in mABDCs as opposed to mLBDCs during differentiation.

### BMP4 elevates alveolar bone formation more than long bone formation

**Figure 5:**
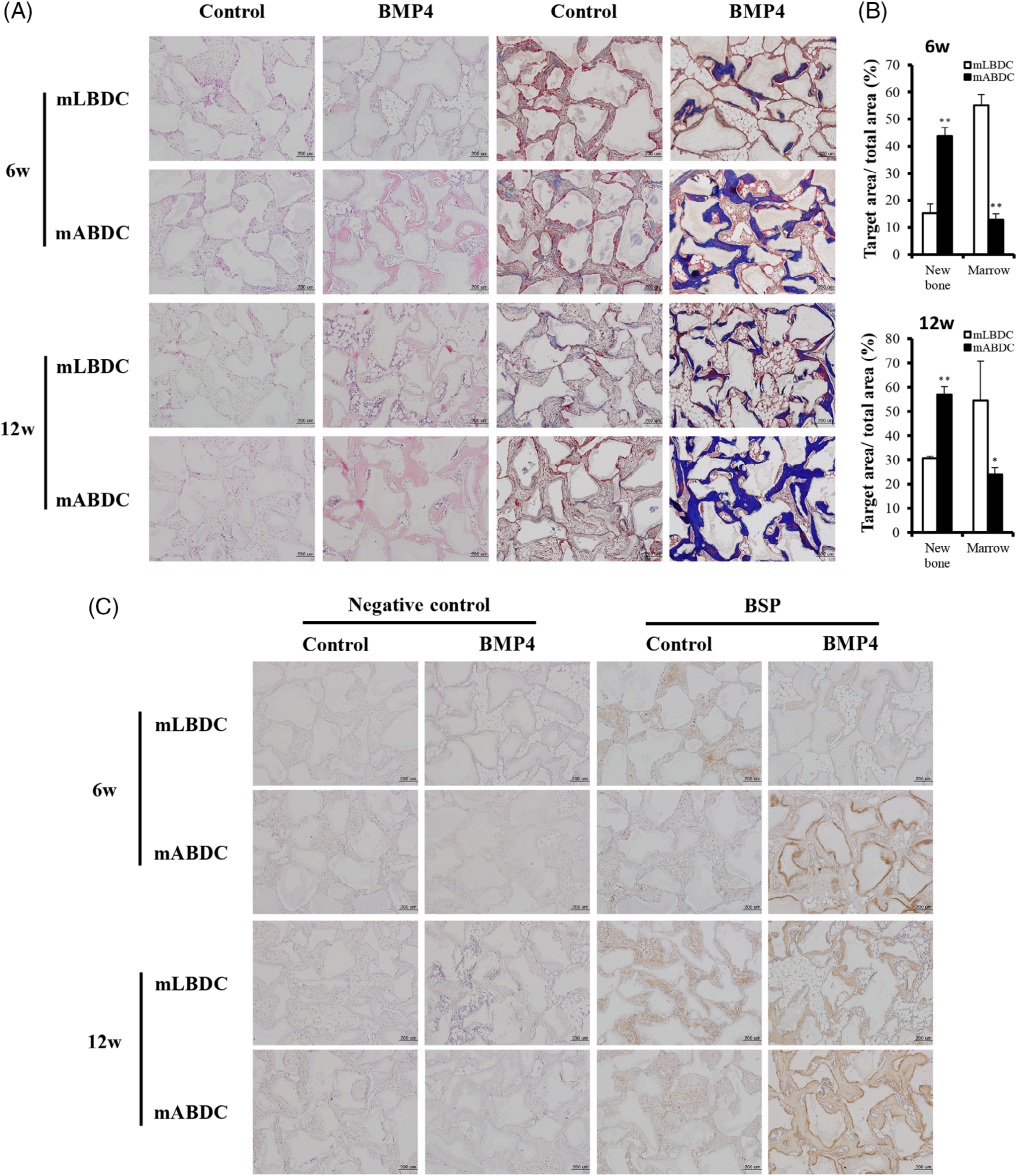
Histological analysis of the regenerated bone matrix using mABDCs and mLBDCs in vivo. The mouse bone‐derived cells were mixed with 100 mg HA/TCP particles alone, or with BMP4 in a 0.5% fibrin gel and transplanted s.c. into immunocompromised mice for 6 and 12 weeks. (*A*) Samples were stained with H&E and Masson's trichrome. (*B*) Quantification of mineralized matrix and marrow‐like space at 6 and 12 weeks. (*C*) Mineralized tissues were immunostained with anti‐bone sialoprotein. Negative control only stained by second rabbit antibody. All statistical analysis performed by Student's *t* test, *n* = 3, *p < 0.05, **p < 0.005. HA/TCP = Hydroxyapatite/tricalcium phosphate; mABDCs = mouse alveolar bone‐derived cells; mLBDCs = mouse long bone‐derived cells.

To determine the role of BMP4 in osteogenic differentiation and bone formation in vivo, we transplanted mABDCs and mLBDCs into subcutaneous tissues of immunocompromised mice in the presence of hydroxyapatite/tricalcium phosphate (HA/TCP) under four different conditions: mABDC‐only, mLBDC‐only, mABDC with rBMP4, and mLBDC with rBMP4. Six weeks after transplantation, bone‐like tissues were formed at the periphery of HA/TCP particles only in BMP4‐treated groups. The rBMP4‐treated mABDC group exhibited more bone‐like tissues than the rBMP4‐treated mLBDC group. Contrastingly, marrow‐like tissue including adipose tissue presented an opposite response. This phenomenon was more prominent after 12 weeks (Fig. 5*A*,*B*). Protein expression of BSP, which is known as a typical bone marker, was higher in the mLBDC‐only group than the mABDC‐only group. Contrarily, mABDCs expressed more BSP protein than mLBDCs in the rBMP4‐treated group (Fig. 5*C*). Osteoclast number was increased in both BMP4‐treated groups (Supplementary Fig. S5). These results confirm that bone formation capacity of BMP4 is greater in mABDCs than in mLBDCs in vivo.

## Discussion

Alveolar bone is a component of the periodontium tissue, which is formed by the ectomesenchyme‐derived dental follicle cell. Some studies reveal that the interaction of ectomesenchyme and dental epithelium is essential during periodontium development, the same goes for tooth development.^(^ ^2^ ^)^ Previous studies mention the beginning stages of mouse alveolar bone formation.^(^ ^19^ ^)^ However, the development stages of alveolar bone are not defined in detail.^(^ ^20^, ^21^, ^22^, ^23^, ^24^ ^)^ For better analysis, the stages were outlined using the tooth‐development stages as a guide. Along with these stages, to compare long to alveolar bone, this study performed a histological analysis. During this analysis, we observed that epithelial tissue was located more closely and for a longer duration to developing alveolar bone than to long bone. From these observations, we infer that alveolar bone formation could be associated with dental epithelium conformational changes. Overall, alveolar bone development could be more related to epithelial tissue than to long bone development.

To exclude as many confounding factors as possible, we harvested mLBDCs from the diaphysis region of the long bone for comparison. There are two kinds of ossification during bone development: intramembranous and endochondral.^(^ ^25^ ^)^ Most craniofacial bones are formed via intramembranous ossification. Long bone is created by endochondral ossification. However, when perichondrium transforms to periosteum, osteoblasts form new bone beneath the perichondrium with intramembranous ossification at the diaphysis region.^(^ ^26^, ^27^ ^)^ By selecting a similarly intramembranous ossified bone, we limited the confounding factors that could arise from comparing differently ossified bone.

To compare alveolar bone to long bone, NC‐ and EMI‐related genes in primary cultured bones between alveolar bone and long bone were analyzed. Primary cultured bone cells showed different characteristics between alveolar bone and long bone in NC‐ and EMI‐related gene expression. Homeobox genes—*Msx* family—are abundant in mesenchymal NC cells in pharyngeal arches and craniofacial skeleton.^(^ ^28^ ^)^ When mutated, mouse exhibited an absence and/or malformation of alveolar bone.^(^ ^29^, ^30^ ^)^ Another NC‐related gene, *Dlx* family genes, also plays a significant role in embryonic development and is seen expressed in cranial NC‐derived craniofacial primordia and limbs. The lack of functional *Dlx5* in mice results in dysmorphogenesis in almost all cranial bones, including incisive and molar alveolar bone.^(^ ^31^ ^)^ However, for limbs, single homozygous mutants of *Msx1, Msx2*, and *Dlx5* do not display gross abnormalities.^(^ ^32^, ^33^ ^)^ As hinted, NC‐related are known as regulators of EMI during orofacial and limb developments.^(^ ^34^ ^)^ NC‐related genes were more highly expressed in alveolar bone tissue and cells than in long bone. Also, their expressions were higher in mABDCs than in mLBDCs during differentiation. These results indicate that an abundant expression of NC‐related genes could be a marker for alveolar bone. We theorized that EMI would affect alveolar bone more than long bone because EMI is strongly regulated from the abundant expression of NC‐related genes.

Previous studies have explored EMI and *Bmp2, Bmp4, Nfic*, or *Cpne7* individually in either alveolar or long bone. In this study, we looked at these individual relationships as a group and attempted to understand how these relationships fit in the bigger picture of how correlated or different alveolar and long bone are. *Bmp* was selected for our analysis because Bmp signaling is crucial for regulating EMI during organogenesis—organs formed include tooth and limb.^(^ ^35^, ^36^ ^)^ Specifically, *Bmp2* and *Bmp4* are frequently codistributed, and their expression shifts between epithelium and mesenchyme.^(^ ^37^ ^)^ Along with *Bmp, Nfic* was also observed because a previous study defined it as a component of EMI in ectomesenchyme.^(^ ^38^ ^)^ *Nfic* KO mice showed defective alveolar bone formation and osteoporosis like phenotype in long bone.^(^ ^39^, ^40^ ^)^ Finally, *Cpne7* was explored between the two because it is secreted from dental epithelium, and its effects on odontogenic induction to dental mesenchyme was confirmed in a previous study.^(^ ^17^, ^41^ ^)^ Although previous studies merely confirmed the existence of EMI in alveolar and long bone, this study illustrated distinct EMI‐related gene expression patterns in alveolar bone and long bone. Upon seeing the expression patterns, we inferred that the regulation of EMI in alveolar bone and long bone could be different.

Along with the differences seen in NC‐ and EMI‐related genes, a difference in mineralization capacity was seen between long and alveolar bone. Mineralization capacity was more elevated in mLBDCs than in mABDCs. In contrast, their morphology and proliferation rate were not significantly different. These results aligned with osteogenic gene expression patterns in mLBDCs through RNA‐seq. Interestingly, a different study showed a more elevated mineralization capacity in mABDCs than in mLBDCs.^(^ ^42^ ^)^ This discrepancy is thought to result from the different bone cell harvesting method. The other study used primary bone marrow cells, whereas we used primary cells from the bone surface.

As mentioned above, although initially inferred, alveolar and long bone showed distinct genetic profiles. Specifically, osteogenic, EMI‐, and NC‐related genes were different in mABDCs and mLBDCs. To confirm these results, RNA sequencing was used, and the difference between a mABDC's and a mLBDC's EMI‐ and NC‐related gene expression was confirmed. Along with the differences seen during standard conditions, EMI‐ and NC‐related gene expression patterns were also different during osteogenic differentiation. Considering how most NC‐related genes are known as transcription factors that regulate osteogenic gene expression, these results hint that different regulation of EMI‐ and NC‐related genes take part in altering bone characteristics to result either in alveolar or long bone.^(^ ^43^ ^)^

To further compare alveolar and long bone, their osteogenic, NC‐related, and EMI‐related gene responsiveness to developing dental epithelial secretions were compared. Their responsiveness to developing dental epithelial secretions was selected because developing dental epithelial secretions induce differentiation of mesenchyme cells through gene regulation.^(^ ^44^ ^)^ To compare the effects, we analyzed gene expression in BMP4‐ and CPNE7‐treated mABDCs and mLBDCs. To recap, despite their correlation as EMI‐related genes, MSX1, MSX2, DLX5, and NFIC were not treated because they are transcription factors inferred to not be secreted from dental epithelial cells.

Initially, osteogenic gene responsiveness to developing dental epithelial secretions was compared between alveolar and long bone. Between BMP4 and CPNE7, BMP4‐treated cells illustrated a stronger regulation of osteogenic genes. BMP4 elevated mRNA expression of osteogenic genes in mABDCs. However, for BMP4‐treated mLBDCs, *OC* was not regulated. Relatively to mLBDCs, *Bsp, Alp*, and *Osx* were more elevated in mABDCs. During differentiation, most osteogenic genes were downregulated in BMP4‐treated mLBDCs. *Bsp* increased in both mABDCs and mLBDCs. The increment of increase in mABDCs after the BMP4 treatment was greater than the magnitude of increase in mLBDCs after the treatment. In the in vivo model—contrary to the cell‐only group—the BMP4‐treated mABDC group formed more BSP‐positive bonelike tissue than BMP4‐treated mLBDCs. Another osteogenic gene and osteocyte marker, *Dmp1*, was upregulated in BMP4‐treated mABDCs at the late stages of differentiation. In mLBDCs, *Dmp1* was downregulated in mLBDCs during the entire differentiation stage. In the in vivo study, Masson's trichrome‐stained tissue showed a more mature bonelike matrix in the BMP4‐treated mABDC group than the BMP4‐treated mLBDC group. Overall, our findings indicate that BMP4 could accelerate the maturation of alveolar bone by stimulating *Dmp1*; this effect was not seen in long bone. Our results show that BMP4 induces more bone formation and maturation in alveolar bone than in long bone by upregulating osteogenic genes.

Following osteogenic gene comparison, the NC‐related gene responsiveness to developing dental epithelial secretions was compared. BMP4, along with the osteogenic genes, was seen to upregulate NC‐related genes, *Msx2* and *Dlx5*, in both cells. *Msx2* gene expression, expressed in mandibular alveolar bone during skeletal growth, was elevated in response to BMP4 during osteogenic differentiation.^(^ ^29^ ^)^ Previous studies show *Msx2* inducing *Alp* activity and inhibiting adipogenesis by diminishing the expression of *Pparγ*.^(^ ^45^ ^)^ Again, when treated with BMP4*, Dlx5* was upregulated in mABDCs during the early stages of differentiation, whereas for mLBDCs, *Dlx5* was downregulated during the entire differentiation stage. There was no change in the other NC‐related gene, *Msx1*. In the in vivo model, volume of marrow‐like tissue including adipose tissue was smaller in BMP4‐treated mABDCs than in BMP4‐treated mLBDCs. BMP4 regulates alveolar bone formation in *Msx1* KO mouse via inducing *Dlx5* and *Runx2* expression.^(^ ^19^ ^)^ These results suggest that BMP4 induces alveolar bone‐specific bone formation by regulating *Msx2* and *Dlx5* expression. Furthermore, although previous studies show that *Msx1* has an effect on BMP4 in alveolar bone, our results show that BMP4 does not affect *Msx1*, inferring a downstream relationship.^(^ ^46^ ^)^ Finally, our results show that elevated levels of *Msx2* could also affect the proportion of marrow tissue in bone.

Finally, for the comparison, EMI‐related gene responsiveness to developing dental epithelial secretions was observed. The responsiveness that was immediately noticed was from *Cpne7, Bmp2*, and *Bmp4*. For CPNE7, we observed that CPNE7 did not regulate any genes in mABDCs and mLBDCs*. Cpne7* was observed to be downregulated by BMP4 in both bone‐derived cells during differentiation. These observations infer that *Cpne7* may have a distinct function in odontogenic cells and osteogenic cells. *Bmp2* is the most well‐known bone‐inducing factor. Interestingly, BMP4 induced *Bmp2* expression in both cells. The rate of increase of *Bmp2* induced by BMP4 was higher in mABDCs than in mLBDCs during differentiation. *Bmp4* expression was downregulated in both BMP4‐treated cells. Based on these results, we inferred the possibility that BMP4 induced bone formation effects resulting from BMP4 upregulating *Bmp2*, which then affects bone formation. Furthermore, we speculated that the effects of BMP4 could be controlled by negative feedback.

While observing the differences between alveolar and long bone, we discovered epithelium and BMP4 potentially affecting alveolar bone. To confirm the previous data regarding NC‐ and EMI‐related gene differences between the two bones, RNA sequencing was performed. When the sequencing was executed, significant differences between the two bones were observed as the following: BMP pathways, BMP responsiveness, odontogenesis, epithelium, and mesenchyme. When the osteogenic gene responsiveness was observed, we saw that BMP4 elevated the mRNA expression of osteogenic genes in mABDCs. Furthermore, when the cell was BMP4 treated, we observed that the rate of increase of *Bsp* was higher in mABDCs than in mLBDCs during differentiation. Regarding the NC‐related gene responsiveness, we saw that BMP4 controlled *Msx2* and *Dlx5* in mesenchyme cells. Although the origin of BMP4 (epithelium or mesenchyme) is unknown, BMP4 seems to be specifically significant in mABDC osteogenic growth, considering how there was a drastic change in EMI‐related gene responsiveness. Overall, once again, EMI‐related gene responsiveness showed that BMP4 could play a significant role in mABDCs by having a steeper increase of BMP4‐induced *Bmp2* in mABDCs than in mLBDCs during differentiation. Our findings line‐up with a previous study that reveals that *Bmp4* knockout in epithelial cells had a more severe effect on alveolar bone than the knockout in mesenchyme cells.^(^ ^22^, ^47^ ^)^ When the damage was compared between alveolar and long bone, the study revealed that damage in alveolar bone was more severe—at times, long bone showed no defect.^(^ ^48^, ^49^ ^)^

In summary, we elucidated the different genetic profiles of alveolar bone and long bone. Additionally, EMI‐related epithelial factor, BMP4, was seen to affect alveolar bone formation more than long bone by regulating the expression of osteogenic, EMI‐, and NC‐related genes. By detailing the differences between alveolar and long bone, the present study emphasizes the need for tissue‐specific bone treatment. This can be seen from the different gene responses for BMP4 in long and alveolar bone. More specifically, based on our results, this study highlights the need of a distinct treatment specifically for alveolar bone, considering how distinctly different the bone is.

## Disclosures

The authors have no conflicts of interest to declare.

## Peer Review

The peer review history for this article is available at https://publons.com/publon/10.1002/jbm4.10382.

## Supporting information


**Fig. S1** Alveolar bone and long bone development during mouse tooth development stage. (A, H) Embryonic day 12 (bud stage). (B, I) Embryonic day 14 (bud‐to‐cap transition). (C, D, J) Embryonic day 16 (2nd molar, cap stage (C) & 1st molar, early bell stage (D)). (E, K) Embryonic day 19 (late bell stage). (F, L) Postnatal day 7 (hard tissue formation). (G, M) Postnatal 21 day (root formation). (A‐G) The region covered by the red dashed line: alveolar bone. The region covered by the black dashed line: basal bone. (H‐M) The region covered by the red dashed line: bone. The region covered by the black dashed line: cartilage. E, embryonic; PN, postnatal.

**Fig. S2** Characteristics of mouse alveolar bone and long bone derived cells. (A‐D) Morphology of cells derived from alveolar bone and long bone. (A, B) Primary cultured cells isolated from alveolar bone pieces and passage 1 cells. (C, D) Primary cultured cells isolated from long bone pieces and passage 1 cells. Cells were observed under an optical microscope (X 100). (E) MTT assay was analyzed in both bone derived cells for 3 days. (F) Expression levels of osteoblast marker gene, Bsp, obtained by real‐time PCR from cDNA of mABDC, mLBDC and mDF. Real‐time PCR values are normalized to the internal housekeeping gene, Gapdh. (G) Alizarin Red S staining (ARS). Cells were cultured in osteogenic induction media for 10 and 20 days.

**Fig. S3** Functional analysis of RNA sequencing data. (A‐F) Bar plot of gene‐enrichment and functional annotation analysis using gene ontology. (A‐D) Terms of biological process category. (A) Development and morphogenesis‐related terms (B) Gene expression and signaling‐related terms. (C) Responsiveness‐related terms. (D) Ossification, differentiation, remodeling, and mineralization‐related terms. (E) Terms of molecular function category. (F) Terms of cellular component category. (G) Top 20 terms in enrichment test of KEGG pathway analysis.

**Fig. S4** Relative mRNA expression of CNC‐related genes. Relative expression of genes expressed in craniofacial NC cells, NC‐derived craniofacial/ pharyngeal arch mesenchyme, craniofacial skeleton, limb bud mesenchyme. Those genes are known as markers of neural crest stem cell, NC progenitor cell, and craniofacial neural crest cell. All statistical analysis performed by Student t‐test, n = 3, *p < 0.05, **p < 0.005.

**Fig. S5** Histological analysis of the osteoclast using mABDC and mLBDC in vivo. Samples were stained with TRAP (Tartrate‐resistant acid phosphatase) (A‐D) mLBDC. (E‐H) mABDC.

**Table S1** Primers used for real‐time PCR.
