# Supplementary figures and images for: Different Responsiveness of Alveolar Bone and Long Bone to Epithelial‐Mesenchymal Interaction‐Related Factor
Source: JBMR Plus. 2020 Jun 21;4(8):e10382. doi: 10.1002/jbm4.10382 (PMC7422712; doi:10.1002/jbm4.10382)

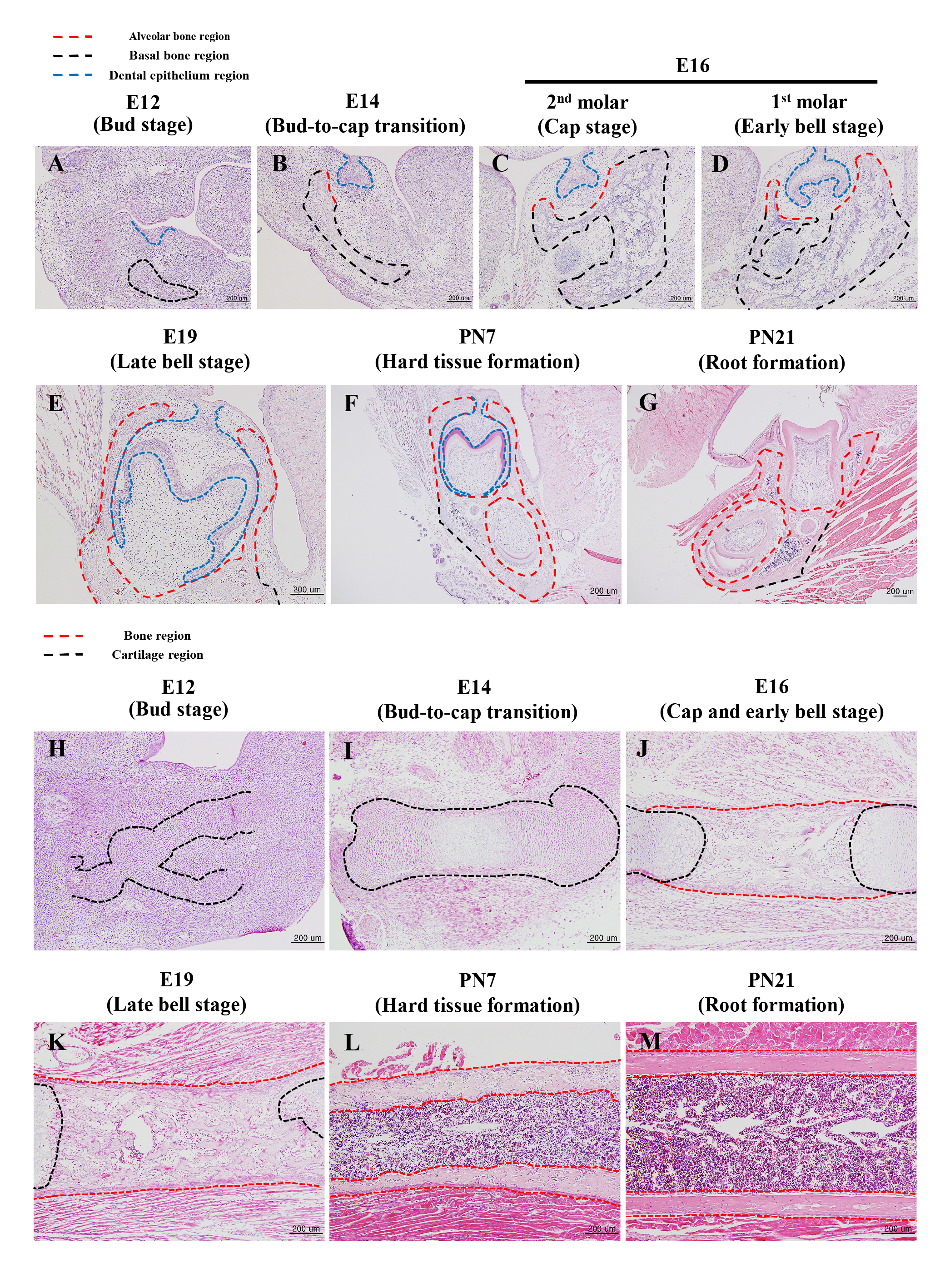

Supplement: Supplementary file 1 — Fig. S1 Alveolar bone and long bone development during mouse tooth development stage. (A, H) Embryonic day 12 (bud stage). (B, I) Embryonic day 14 (bud‐to‐cap transition). (C, D, J) Embryonic day 16 (2nd molar, cap stage (C) & 1st molar, early bell stage (D)). (E, K) Embryonic day 19 (late bell stage). (F, L) Postnatal day 7 (hard tissue formation). (G, M) Postnatal 21 day (root formation). (A‐G) The region covered by the red dashed line: alveolar bone. The region covered by the black dashed line: basal bone. (H‐M) The region covered by the red dashed line: bone. The region covered by the black dashed line: cartilage. E, embryonic; PN, postnatal. [file JBM4-4-e10382-s001.tif]

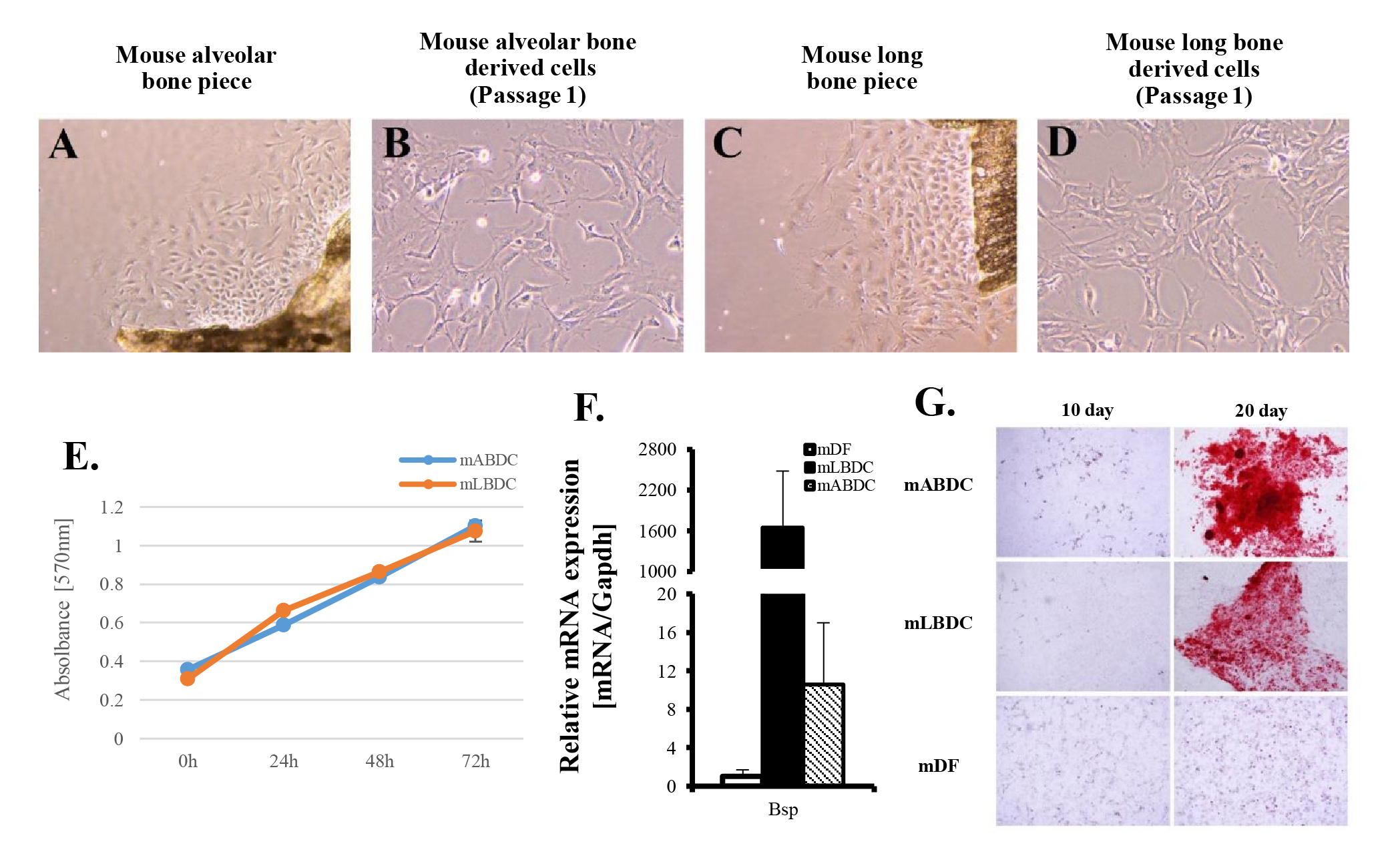

Supplement: Supplementary file 2 — Fig. S2 Characteristics of mouse alveolar bone and long bone derived cells. (A‐D) Morphology of cells derived from alveolar bone and long bone. (A, B) Primary cultured cells isolated from alveolar bone pieces and passage 1 cells. (C, D) Primary cultured cells isolated from long bone pieces and passage 1 cells. Cells were observed under an optical microscope (X 100). (E) MTT assay was analyzed in both bone derived cells for 3 days. (F) Expression levels of osteoblast marker gene, Bsp, obtained by real‐time PCR from cDNA of mABDC, mLBDC and mDF. Real‐time PCR values are normalized to the internal housekeeping gene, Gapdh. (G) Alizarin Red S staining (ARS). Cells were cultured in osteogenic induction media for 10 and 20 days. [file JBM4-4-e10382-s002.tif]

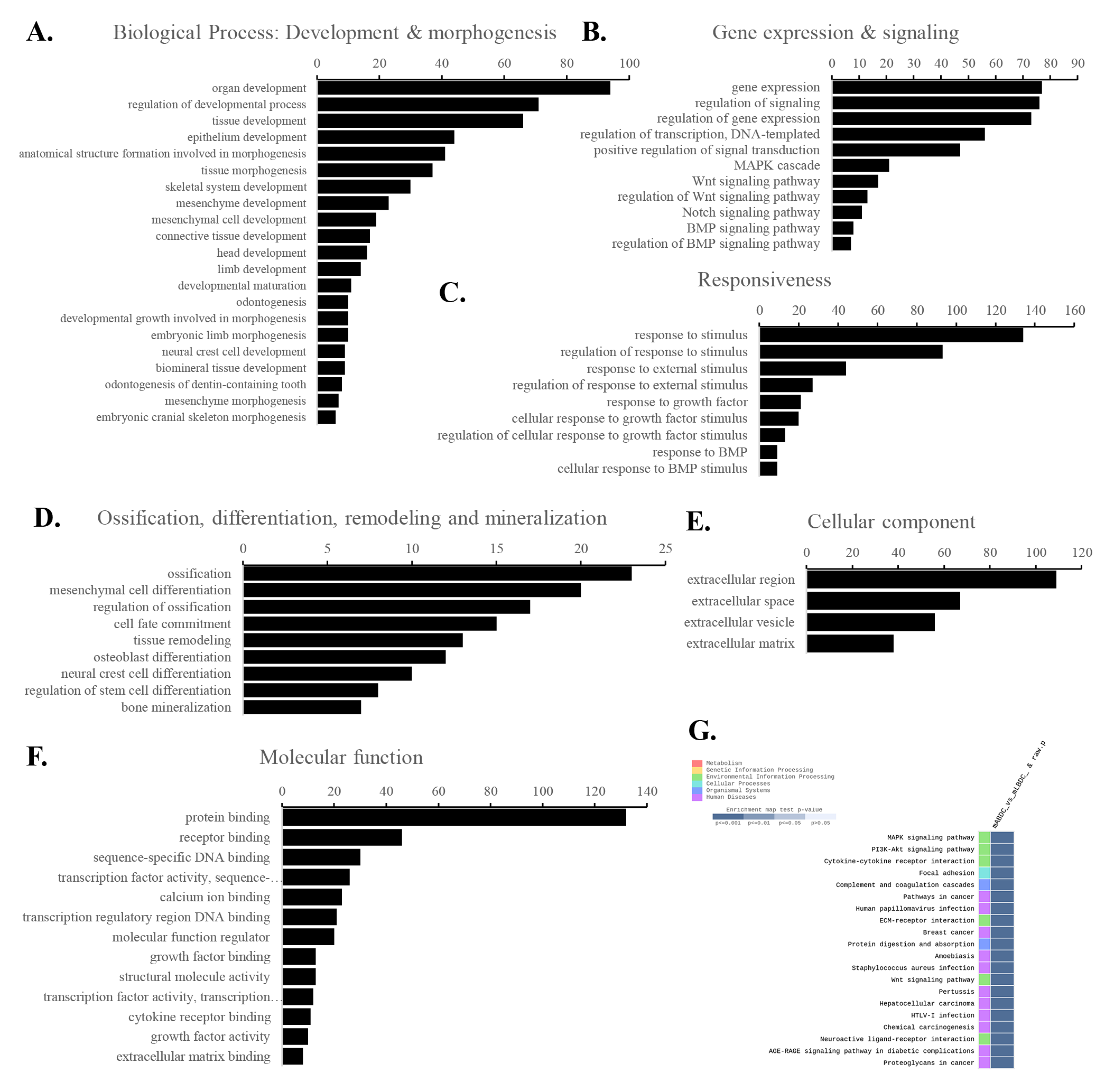

Supplement: Supplementary file 3 — Fig. S3 Functional analysis of RNA sequencing data. (A‐F) Bar plot of gene‐enrichment and functional annotation analysis using gene ontology. (A‐D) Terms of biological process category. (A) Development and morphogenesis‐related terms (B) Gene expression and signaling‐related terms. (C) Responsiveness‐related terms. (D) Ossification, differentiation, remodeling, and mineralization‐related terms. (E) Terms of molecular function category. (F) Terms of cellular component category. (G) Top 20 terms in enrichment test of KEGG pathway analysis. [file JBM4-4-e10382-s003.tif]

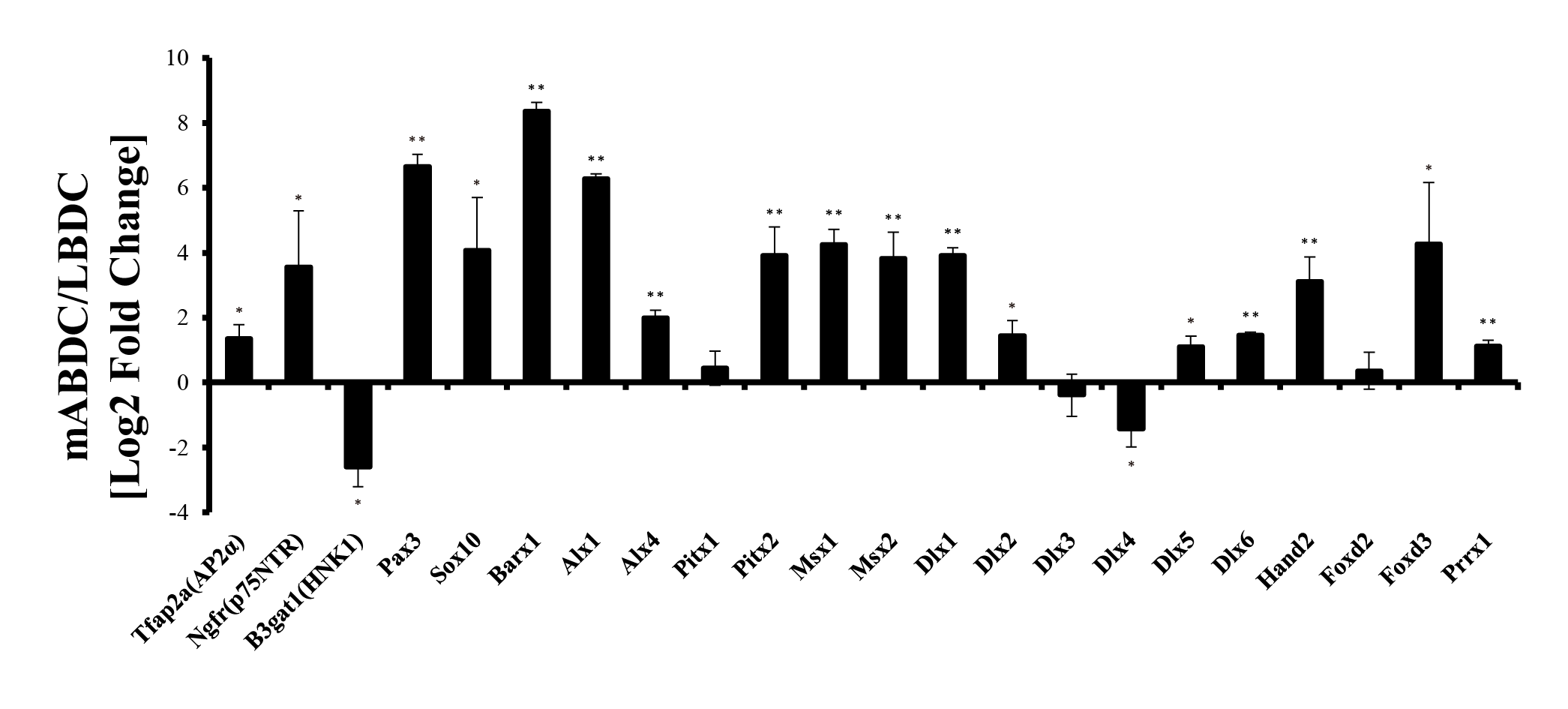

Supplement: Supplementary file 4 — Fig. S4 Relative mRNA expression of CNC‐related genes. Relative expression of genes expressed in craniofacial NC cells, NC‐derived craniofacial/ pharyngeal arch mesenchyme, craniofacial skeleton, limb bud mesenchyme. Those genes are known as markers of neural crest stem cell, NC progenitor cell, and craniofacial neural crest cell. All statistical analysis performed by Student t‐test, n = 3, *p < 0.05, **p < 0.005. [file JBM4-4-e10382-s004.tif]

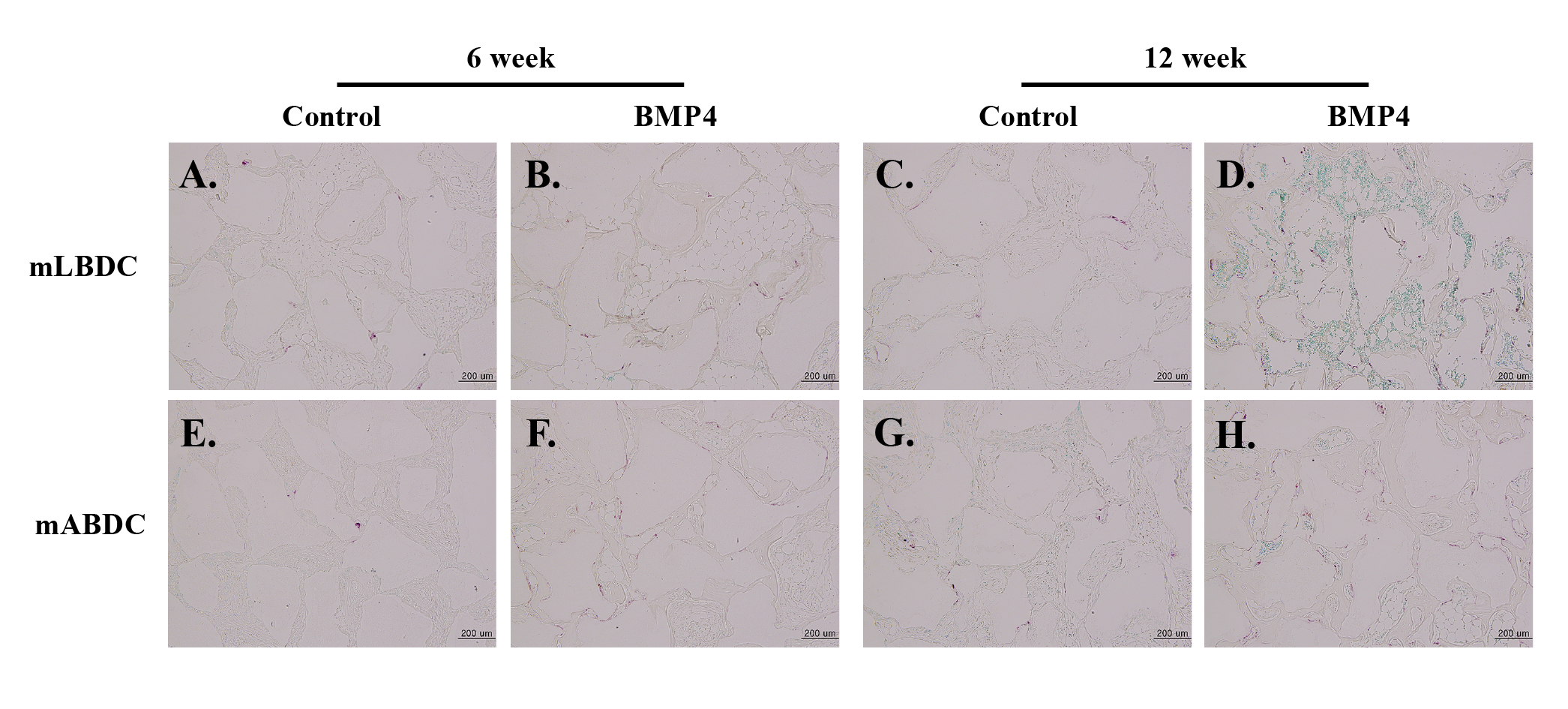

Supplement: Supplementary file 5 — Fig. S5 Histological analysis of the osteoclast using mABDC and mLBDC in vivo. Samples were stained with TRAP (Tartrate‐resistant acid phosphatase) (A‐D) mLBDC. (E‐H) mABDC. [file JBM4-4-e10382-s005.tif]
