# Supplementary material for: Different Responsiveness of Alveolar Bone and Long Bone to Epithelial‐Mesenchymal Interaction‐Related Factor
Source: JBMR Plus. 2020 Jun 21;4(8):e10382. doi: 10.1002/jbm4.10382 (PMC7422712; doi:10.1002/jbm4.10382)
Supplement: Supplementary file 6 — Table S1 Primers used for real‐time PCR. [file JBM4-4-e10382-s006.docx]

**Supplementary Table 1.** Primers used for real-time PCR.

| **Genes** | **Primers** |
| --- | --- |
| *mGapdh* | F 5’-AGGTCGGTGTGAACGGATTTG-3’  R 5’-TGTAGACCATGTAGTTGAGGTCA-3’ |
| *mBsp* | F 5’-CCGGCCACGCTACTTTCTT-3’  R 5’-TGGACTGGAAACCGTTTCAGA-3’ |
| *mOC* | F 5’-CTGACAAAGCCTTCATGTCCAA-3’  R 5’-GCGCCGGAGTCTGTTCACTA-3’ |
| *mAlp* | F 5’-CCAACTCTTTTGTGCCAGAGA-3’  R 5’-GGCTACATTGGTGTTGAGCTTTT-3’ |
| *mRunx2* | F 5’-TTCTCCAACCCACGAATGCAC-3’  R 5’-CAGGTACGTGTGGTAGTGAGT-3’ |
| *mOsx* | F 5’-CCCACCCTTCCCTCACTCAT-3’  R 5’-CCTTGTACCACGAGCCATAGG-3’ |
| *mDmp1* | F 5’-CATTCTCCTTGTGTTCCTTTGGG-3’  R 5’-TGTGGTCACTATTTGCCTGTC-3’ |
| *mMsx1* | F 5’-GAAACTAGATCGGACCCCGTGGAT-3’  R 5’-GCTTGCGGTTGGTCTTGTGCTT-3’ |
| *mMsx2* | F 5’-TTCACCACATCCCAGCTTCTA-3’  R 5’-TTGCAGTCTTTTCGCCTTAGC-3’ |
| *mDlx5* | F 5’-CTGGCCGCTTTACAGAGAAG-3’  R 5’-TCACCTGTGTTTGCGTCAGT-3’ |
| *mBmp2* | F 5’-GGGACCCGCTGTCTTCTAGT-3’  R 5’-TCAACTCAAATTCGCTGAGGAC-3’ |
| *mBmp4* | F 5’-TTCCTGGTAACCGAATGCTGA-3’  R 5’-CCTGAATCTCGGCGACTTTTT-3’ |
| *mNfic* | F 5’-GACCTGTACCTGGCCTACTTTG-3’  R 5’-CACACCTGACGTGACAAAGCTC-3’ |
| *mCpne7* | F 5’-CGGGACCCATTGACCAAGTC-3’  R 5’-CATACACCTCAAACCGTAGCTTC-3’ |
